# Circulating hsa-miR-323b-3p in Huntington's Disease: A Pilot Study

**DOI:** 10.3389/fneur.2021.657973

**Published:** 2021-05-05

**Authors:** Michela Ferraldeschi, Silvia Romano, Simona Giglio, Carmela Romano, Emanuele Morena, Rosella Mechelli, Viviana Annibali, Martina Ubaldi, Maria Chiara Buscarinu, Renato Umeton, Gabriele Sani, Andrea Vecchione, Marco Salvetti, Giovanni Ristori

**Affiliations:** ^1^Ospedale San Giovanni Battista, ACISMOM, Rome, Italy; ^2^Department of Neurosciences, Centre for Experimental Neurological Therapies (CENTERS), Mental Health and Sensory Organs, Sapienza University of Rome, Rome, Italy; ^3^Department of Experimental Medicine, Policlinico Umberto i of Rome, Sapienza University, Rome, Italy; ^4^Istituti di Ricovero e Cura a Carattere Scientifico San Raffaele Pisana, San Raffaele Roma Open University, Rome, Italy; ^5^Department of Informatics and Analytics, Dana-Farber Cancer Institute, Boston, MA, United States; ^6^Massachusetts Institute of Technology, Cambridge, MA, United States; ^7^Harvard School of Public Health, Boston, MA, United States; ^8^Weill Cornell Medicine, New York City, NY, United States; ^9^Section of Psychiatry, Department of Neuroscience, University Cattolica del Sacro Cuore, Rome, Italy; ^10^Department of Psychiatry, Fondazione Policlinico Agostino Gemelli Istituti di Ricovero e Cura a Carattere Scientifico, Rome, Italy; ^11^Surgical Pathology Units, Department of Clinical and Molecular Medicine, Ospedale Sant'Andrea, Sapienza University, Rome, Italy; ^12^Istituti di Ricovero e Cura a Carattere Scientifico Istituto Neurologico Mediterraneo (INM) Neuromed, Pozzilli, Italy; ^13^Neuroimmunology Unit, Istituti di Ricovero e Cura a Carattere Scientifico Fondazione Santa Lucia, Rome, Italy

**Keywords:** circulating miRNA, huntington disease, biomarkers, digital droplet PCR, bioinformatic analysis

## Abstract

The momentum of gene therapy in Huntington's disease (HD) deserves biomarkers from easily accessible fluid. We planned a study to verify whether plasma miRNome may provide useful peripheral “reporter(s)” for the management of HD patients. We performed an exploratory microarray study of whole non-coding RNA profiles in plasma from nine patients with HD and 13 matched controls [eight healthy subjects (HS) and five psychiatric patients (PP) to minimize possible iatrogenic impact on the profile of non-coding RNAs]. We found an HD-specific signature: downregulation of hsa-miR-98 (fold change, −1.5, *p* = 0.0338 HD vs. HS, and fold change, 1.5, *p* = 0.0045 HD vs. PP) and upregulation of hsa-miR-323b-3p (fold change, 1.5, *p* = 0.0007 HD vs. HS, and fold change, 1.5, *p* = 0.0111 HD vs. PP). To validate this result in an independent cohort, we quantify by digital droplet PCR (ddPCR) the presence of the two microRNA in the plasma of 33 HD patients and 49 matched controls (25 HS and 24 PP patients). We were able to confirm that hsa-miR-323b-3p was upregulated in HD and premanifest HD vs. HS and PP: the median values (first–third quartile) were 4.1 (0.9–10.53) and 5.8 (1.9–10.70) vs. 0.69 (0.3–2.75) and 1.4 (0.78–2.70), respectively, *p* < 0.05. No significant difference was found for hsa-miR-98. To evaluate the biological plausibility of the hsa-miR-323b-3p as a component of the disease pathophysiology, we performed a bioinformatic analysis based on its targetome and the huntingtin (HTT) interactome. We found a statistically significant overconnectivity between the targetome of hsa-miR-323b-3p and the HTT interactome (*p* = 1.48e−08). Furthermore, there was a significant transcription regulation of the HTT interactome by the miR-323b-3p targetome (*p* = 0.02). The availability of handy, reproducible, and minimally invasive biomarkers coming from peripheral miRNome may be valuable to characterize the illness progression, to indicate new therapeutic targets, and to monitor the effect of disease-modifying treatments. Our data deserve further studies with larger sample size and longitudinal design.

## Introduction

Huntington's disease (HD) is an autosomal dominant disease caused by a cytosine, adenine, and guanine (CAG) repeat expansion in the gene that encodes huntingtin (HTT). Although HTT is ubiquitously expressed, patients with HD show predominantly central nervous system (CNS) manifestations, including abnormal motor symptoms, cognitive decline, and psychiatric disturbances. The momentum of gene therapy approaches, in progress or in preparation, calls for informative biomarkers capable of predicting the disease progression and the therapeutic response and driving possible “preventive treatment” in the prodromal phase of the disease (1). The initial attempts focused on cognitive, motor, and neuroimaging biomarkers (2–4), which are more useful in subjects with overt disease, followed at centers with high expertise and dedicated neuroradiological unit.

To identify handy and easily quantifiable biomarkers, biofluids were considered, in particular cerebrospinal fluid (CSF), as a proxy of CNS pathophysiology: mutant HTT was quantified in CSF and was used as a biomarker of effectiveness in the first study of gene therapy in HD patients (5). Following approaches focused on more accessible fluids, such as blood, and several studies reported informative results: plasma levels of 24S-hydroxychiolesterol (6, 7), mitochondrial DNA (mtDNA) (8), and neurofilament light protein (NFL) were described as potential biomarkers in HD. Especially NFL resulted a reliable biomarker of axonal damage with a good correlation between CSF and blood concentrations (9), even though a recent report showed that CSF NFL appeared to be more sensitive than plasma NFL in monitoring disease progression (10). The peripheral blood mononuclear cells (PBMCs) from HD patients were recently used to study the mutant HTT (mHTT) (11), gene expression profiles (12), the length of telomers (13), and the DNA damage response (14), reporting promising results to implement future peripheral biomarkers.

MicroRNAs (miRNAs) are a class of non-coding RNAs that represent a major system of posttranscriptional regulation, by either preventing translational initiation or by targeting transcripts for storage or for degradation. These single-stranded, 19–25 nucleotide-long RNAs are abundant in the CNS and assist in various neuronal processes such as synaptic development, maturation, and plasticity (15, 16). Several studies on microRNA pathogenic role in HD have been published in the last two decades, especially in postmortem analyses: besides the dysregulation of single micro-RNA, such as miR-9/9^*^, miR-132, miR-4488, miR-196a-5p, and miR-549a, among others (17–22), the alteration of the miRNome in HD CNS seems to be global because the mHTT affects the action of argonaute-2, potentially interfering with the biogenesis of all micro-RNA (23). Micro-RNA levels have also been measured in biofluids from HD patients, such as CSF (24), PBMC (25), and plasma (20, 26–28). Previous results on circulating microRNAs are still preliminary, rather inconsistent, and have been obtained on small populations. We therefore planned a study with a stringent experimental setting to verify whether and at what extent peripheral miRNome may provide useful “reporter(s)” for the management of HD patients.

## Materials and Methods

### Study Population

Participants in the study were consecutively enrolled at the Center for Experimental Neurological Therapies, Unit of Neurology (patients with positive test for HD and healthy subjects) and at the School of Medicine and Psychology, Unit of Psychiatry (patients with psychiatric disorders), S. Andrea Hospital, Department of Neurosciences, Mental Health, and Sensory Organs, Sapienza University of Rome, Italy. The study was approved by the local Ethics Committee, and a written consent was obtained from all participants. The operators were unaware of the disease state of each sample during processing and statistical analysis. Eligible subjects for this study were patients with positive genetic test for HD, healthy subjects, and patients with diagnosis of schizophrenia or bipolar disorders under antipsychotic drugs. Exclusion criteria were pregnancy, breastfeeding, and severe systemic illnesses or conditions.

### Plasma Preparation and Affymetrix Gene Chip miRNA Array

Blood samples were obtained by venous punctures in ethylenediaminetetraacetic acid (EDTA) tubes for plasma preparation. Plasma was obtained by centrifugation (1,500 *g* for 15 min at 4°C), and 200 μl of supernatant was stored at −80°C. RNA was extracted using Plasma/Serum Circulating RNA Purification Kit (NORGEN) following the manufacturer's instructions. RNA quality and purity were assessed with the use of the RNA 6000 Nano Assay on Agilent 2100 Bioanalyzer (Agilent). Briefly, 500 ng of total RNA was labeled using FlashTag Biotin HSR (Genisphere LLC) and hybridized to GeneChip® miRNA 2.0 Arrays. The arrays were stained in the Fluidics Station 450 and then scanned on the GeneChip® Scanner 3000 (Affymetrix, USA). The statistical analysis was performed by Transcriptome Analysis Console (TAC) software (Thermo Fisher Scientific). In order to survey the presence of outliers that could disturb the dataset, a principal component analysis (implemented by means of R statistical software) was performed to identify possible subjects needed to be excluded from the dataset. MicroRNA probe outliers were defined from the manufacturer's instructions (Affymetrix), and further analysis included data summarization, normalization, and quality control using the web-based miRNA QC Tool software (Affymetrix). The raw dataset is available in the Gene Expression Omnibus (GEO) repository (GSE167630).

### Digital Droplet PCR

RNAs extraction was performed using miRNeasy Serum/Plasma kit according to the manufacturer's protocol (Qiagen). Micro-RNAs analysis (for hsa-miR-98 and hsa-miR-323b-3p) was performed by digital droplet PCR (ddPCR) (Bio-Rad) using predesigned TaqMan® MicroRNA Assay (Life Technologies) according to the manufacturer's instructions. The volume of 1.5 μl RNA was reversely transcribed to complementary DNA (cDNA) with miRNA-specific primers for hsa-miR-98 and hsa-miR-323b-3p, respectively, in a 15-μl reaction mixture. Twenty microliters of the reaction mixture containing 5 μl of the RT product, 10 μl of Digital PCR Supermix™ (Bio-Rad), and 1 μl of TaqMan primer/probe mix (Applied Biosystems) and diethylpyrocarbonate (DEPC) water were loaded into a plastic cartridge with 70 μl of QX100 Droplet Generation oil and then were placed into the QX100 Droplet Generator (Bio-Rad). The droplets generated from each sample were transferred to a 96-well PCR plate (Eppendorf, Germany). PCR amplification was carried on a T100 thermal cycler (Applied Biosystems) at 95°C for 10 min, followed by 40 cycles of 95°C for 30 s and 60°C for 1 min, then 1 cycle of 98°C for 10 min, ending at 4°C. The plate was loaded on Droplet Reader (Bio-Rad) and read automatically. Absolute quantification of each miRNA was calculated from the number of positive counts per panel using the Poisson distribution. The quantification of the target miRNAs was presented as the number of copies per microliter of PCR mixture.

### Bioinformatic and Statistical Analyses

miRNA target set (i.e., targetome; Supplementary Datasheet 1) was extracted from miRDB (29) *via* the website: https://mirdb.org/cgi-bin/search.cgi?searchType=miRNA&searchBox=hsa-miR-323b-3p&full=1. Overconnectivity (method summary is available at the website: https://portal.genego.com/help2/wwhelp/wwhimpl/js/html/wwhelp.htm#href=MetaCore/MetaCore%20Help.1.245.html) and transcription–regulation analyses (method detail at the website: https://portal.genego.com/help2/wwhelp/wwhimpl/js/html/wwhelp.htm#href=MetaCore/MetaCore%20Help.1.246.html#9006259) were performed using MetaCore v. 20.1 build 70000. The HTT interactome was extracted from BioGRID (30) *via* the website: https://thebiogrid.org/downloadgenerator.php?fileid=143b00f05c23f53fcaab95cb1d2fd584 (Supplementary Datasheet 2).

Raw data were analyzed using Partek Genomic Suite software. Looking for differently expressed non-coding RNAs, we found a candidate HD-specific set compared to both control groups (HS and PP) (∣fold changes∣>1.5, *p* < 0.05 with false discovery rate (FDR) 0.25). Differences in miRs levels between groups were assessed using Mann–Whitney test because of a non-normal distribution of the data. We used medians with interquartile ranges and means ± standard deviations for data presentation. Receiver operating characteristic (ROC) curves were created and the area under the curve (AUC) calculated to evaluate the miRNA classifier capacity for detecting HD. Statistically significant *p*-values were set at *p* < 0.05. All tests were two-sided. The analyses were performed using R (The R Project for Statistical Computing) v3.6 and GraphPad Prism v.5.

## Results

**Figure 1:**
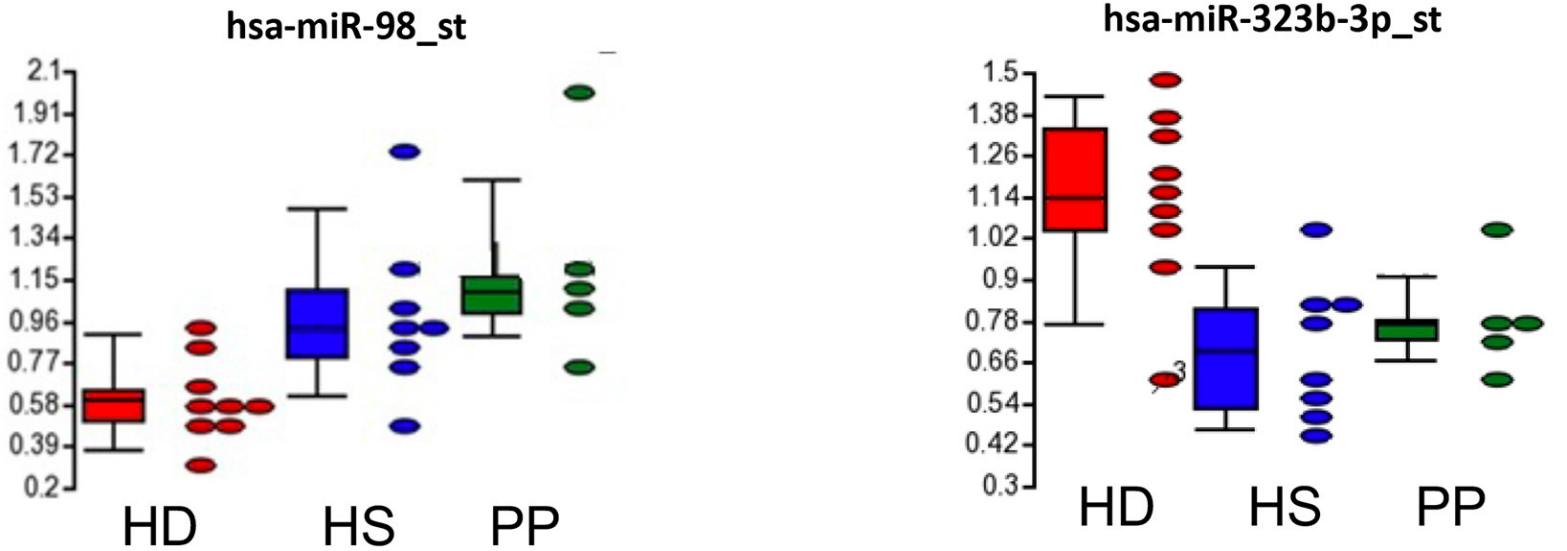
HD-specific signature of circulating miRNA in HD subjects with respect to control groups (HS, healthy subjects; PP, psychiatric patients).

We performed an exploratory microarray study of whole non-coding RNA expression profiles in plasma from 9 patients with Huntington's disease (HD; 5 male and 4 female, mean age of 48.25 ± 10.47) and 13 controls, including 8 healthy subjects (HS; 4 male and 4 female; mean age of 49.17 ± 11.79) and 5 psychiatric patients (PP; 3 male and 2 female; mean age of 50.25 ± 11.47), with schizophrenia or bipolar disorder (see demographic characteristics of each group in Supplementary Table 1). The HD patients were under antipsychotic drugs, the most effective treatment option also in PP (we included in the study the PP control group to minimize a possible iatrogenic impact of neuroleptic drugs on the profile of non-coding RNAs). We found an HD-specific signature: downregulation of hsa-miR-98 (fold change, −1.5, *p* = 0.0338 HD vs. HS and fold change, −1.5, *p* = 0.0045 HD vs. PP) and upregulation of hsa-miR-323b-3p in HD subjects with respect to control groups (fold change, 1.5, *p* = 0.0007 HD vs. HS, and fold change, 1.5, *p* = 0.0111 HD vs. PP; Figure 1).

**Figure 2:**
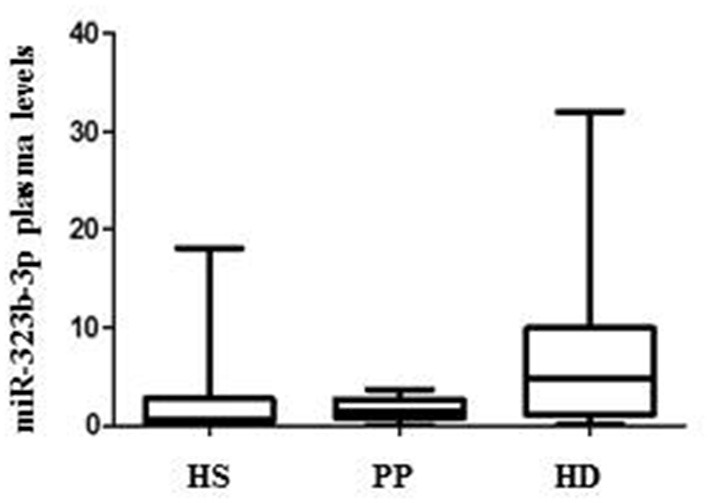
Differential plasma levels of miR-323b-3p in Huntington's disease (HD), healthy subjects (HS), and psychiatric patients (PP). Values are expressed as copies/μl. The horizontal line in the middle of each box indicate the median, whereas the top and bottom black lines the 75th and 25th percentiles, respectively. Mann–Whitney U test. *p* < 0.05.

To validate this result in an independent cohort, we quantify by ddPCR the presence of the two microRNA in the plasma of 33 mHHT carriers [mean age, 55.09 ± 13; 18 male and 15 female; mean Unified Huntington's Disease Rating Scale (UHDRS) (1), 23.4 ± 18.5; mean CGA repeats, 42.88 ± 2), 25 HS (mean age, 51.3 ± 12.4; 11 male and 14 female), and 24 PP patients (mean age, 48.3 ± 14; 14 male and 10 female; Table 1). We were able to confirm that hsa-miR-323b-3p was upregulated in HD compared to control groups (Figure 2), while no significant difference was found for hsa-miR-98. No difference emerged for any microRNA between the two control groups (HS vs. PP). When the mHTT carriers were divided between manifest and premanifest HD, both groups showed significant upregulation of hsa-miR-323b-3p compared to HS and PP, despite the small number of subjects with prodromal disease (*n* = 7). The median values (first to third quartile) in HD and premanifest HD were 4.1 (0.9–10.53) and 5.8 (1.9–10.70) vs. 0.69 (0.3–2.75) and 1.4 (0.78–2.70) in HS and PP, respectively (*p* < 0.05; there was no significant difference between premanifests and HD patients; Table 2).

**Table 1:** Characteristics of HD patients and controls.

	**mHTT carriers (7 pre-HD; 26 HD patients)**	**Healthy subjects (25)**	**Psychiatric patients (24)**
Age, years Mean ± SD	55.1 ± 13	51.3 ± 12.4	48.3 ± 14
M/F	18/15	11/14	14/10
UHDRS-TMS Mean ± SD	24.4 ± 18.65		
CAG Mean ± SD	42.88 ± 2		
Disease duration Mean ± SD	6.3 ± 4.28		

*HD, Huntington's disease; mHTT, mutant huntingtin; pre-HD, individuals in the prodromal phase of the disease; M, male; F, female; UHDRS-TMS, Unified Huntington's Disease Rating Scale-Total Motor Score; CAG, cytosine, adenine, and guanine; SD, standard deviation*.

**Table 2:** Plasma levels of miR-323b-3p in mHTT carriers and control groups.

**Sample (*n*)**	**miR-323b-3p (median, 1st and 3rd quartiles)**	***P* vs. healthy subjects**
Healthy subjects (25)	0.69 (0.30–2.75)	–
Premanifest-HD (7)	5.8 (1.90–10.70)	0.017
Manifest HD (26)	4.1 (0.90–10.53)	0.018
Psychiatric patients (24)	1.4 (0.78–2.70)	0.42

The potential of hsa-miR-323b-3p to distinguish HD group and controls was investigating using ddPCR values in an ROC curve analysis. We observed a trend in predictive capability when using a univariate logistic regression model that used the hsa-miR-323b-3p value to predict the disease class (mHTT carriers vs. controls; Supplementary Table 2). When using 3 × cross-validation and with multiple data splicing initializations, we saw a large variability in sensitivity and AUC, most likely due to the low sample size. We did not find correlations between hsa-miR-323b-3p levels and disease duration, UHDRS, or age at onset.

To evaluate the biological plausibility of the hsa-miR-323b-3p as an actor of the disease pathophysiology, we performed a bioinformatic analysis based on its targetome (Supplementary Datasheet 1) and the HTT interactome (Supplementary Datasheet 2). We found a statistically significant overconnectivity between the targetome of hsa-miR-323b-3p and the HTT interactome (*p* = 1.48e−08), as well as a significant transcription regulation of the HTT interactome by the miR-323b-3p targetome (*p* = 0.02; Table 3).

**Table 3:** Overconnectivity analysis (all interactions and transcription regulation only) between two sets of genes.

**Set 1**	**Set 2**	**Interactions considered**	Actual^*^	R^*^	n^*^	N^*^	Exp^*^	Ratio^*^	z score^*^	*P*-value^*^
miR-323b-3p targets	HTT interactome	All interactions from Set 1 to Set 2 and from Set 2 to Set 1	2,516	41,663	33,446	615,471	2,264	1.111	5.639	1.480E−08
miR-323b-3p targets	HTT interactome	Only transcription regulation of items in Set 2 by items in Set 1	146	6,965	3,689	208,888	123	1.187	2.128	0.02

* Abbreviations: Actual, number of links between sets; n, number of incoming links between sets; R, number of outgoing links between sets; N, total number of links in the complete database or background list; Exp(ected), mean value for hypergeometric distribution (n*R/N); Ratio, connectivity ratio (Actual/Expected); z score = [(actual–expected)/sqrt(variance)]; p-value probability to have the given value of actual or higher (or lower for negative z score).

## Discussion

This pilot study showed that hsa-miR-323b-3p is upregulated in individuals carrying mHTT. The result was obtained in the context of a stringent experimental setting: a two-stage analysis of circulating microRNA through an unbiased microarray approach, followed by a quantitative measurement by ddPCR; a control for the potential confounding treatment effects, through the inclusion of a matched population of PP taking drug classes often prescribed also to HD patients. The narrow range of triplet repeats (see in section Results) adds another component of stringency (a homogeneous patient population that minimizes variability inside HD group).

During the last two decades, several studies were aimed at clarifying the role of human micro-RNAs in HD, especially in a neuropathological context (23). However, a role for hsa-miR-323b-3p has never been reported. The data coming from our overconnectivity analysis between hsa-miR-323b-3p targetome and HTT interactome suggest a possible role of this microRNA in the disease pathogenesis. The small RNAs have been identified as highly stable in the biofluids (31), being potential “reporters” of pathogenic loop occurring in the CNS and representing also potential therapeutic targets to break off detrimental molecular cascades triggered by mHTT. Our study adds to other works (20, 26–28) on the potential utility of circulating microRNAs, as mirrors of the pathogenic processes that take place in the CNS (18, 20), as well as biomarkers showing quantifiable changes after symptomatic approaches (28). Moreover, circulating micro-RNAs may offer potential advantages in terms of disease specificity compared to other approach, such as circulating NFL, which was already shown to be a promising peripheral biomarker for many neurological diseases, including HD (32, 33).

The limitations of the present work fall within those of the pilot study. The sample size was informative although not balanced between premanifest carriers of HTT mutation and individuals with overt disease. The focus on the narrow range of HTT expansion length could limit generalizability of the findings. Future works on a larger sample of cases will allow to overcome such limitation. Moreover, longitudinal data showing the dynamics of the hsa-miR-323b-3p over time are missing in both patients and premanifest HD. These limitations may, at least in part, account for the lack of correlation between the hsa-miR-323b-3p blood levels and measures of disease progression. In fact, a trend (*p* = 0.09) toward an inverse relationship between disease duration and has-miR-323b-3p values emerged (Supplementary Figure 1). These data, together with the difference in the micro-RNA median values between overt and premanifest HD (respectively, 4.1 vs, 5.8), suggest highest values of circulating has-miR-323b-3p in prodromal disease, followed by a decrease at still higher-than-normal level in the overt disease. This dynamics may be reminiscent of our recent studies on a biomarker based on the damage DNA response in peripheral leukocytes of prodromal HD (14). Future longitudinal studies with repeated assays in the same subjects and an adequate number of premanifest HD carriers will allow to validate the possible use of has-miR-323b-3p as an informative predictor of disease evolution. This work does not allow to benchmark hsa-miR-323b-3p against circulating NFL or CSF mHTT (that are reported as informative biomarkers in biofluids from HD patients) because we did not assay them in this pilot study.

The value of the present study stems from its timeliness, in a landscape of HD management that is rapidly evolving toward etiological therapies. The availability of handy, reproducible, and minimally invasive biomarkers, coming from peripheral blood and easier to obtain than other predictors, may be valuable to characterize the illness progression, to indicate new therapeutic targets, and to monitor the effect of disease-modifying treatments. Future personalized therapies for different groups of HD patients and possible trials with etiological approaches in premanifest subjects might pose novel needs, which will plausibly take advantages from circulating non-coding RNAs as peripheral biomarkers.

## Data Availability Statement

The datasets presented in this study can be found in online repositories. The names of the repository/repositories and accession number(s) can be found here: GEO, GSE167630.

## Ethics Statement

The studies involving human participants were reviewed and approved by Azienda ospedaliera Sant andrea, Sapienza Università di roma. The patients/participants provided their written informed consent to participate in this study.

## Author Contributions

SR and MF were the study principal investigators. MF, SR, VA, GR, GS, and MS conceived and designed the study. MF, SR, MB, EM, CR, and MU were assessing neurologists. RU provided bioinformatic analyses. VA, AV, and SG acquired and analyzed data on miRNA. SR and SG performed the statistical analyses. GR, SR, MF, and MS contributed to write the manuscript. All the authors were involved in the assessment and interpretation of the data.

## Conflict of Interest

The authors declare that the research was conducted in the absence of any commercial or financial relationships that could be construed as a potential conflict of interest.
