# Supplementary material for: Circulating hsa-miR-323b-3p in Huntington's Disease: A Pilot Study
Source: Front Neurol. 2021 May 5;12:657973. doi: 10.3389/fneur.2021.657973 (PMC8131841; doi:10.3389/fneur.2021.657973)
Supplement: Supplementary file 1 [file Table_1.doc]

**Supplementary table 1. Characteristics of HD patients and controls of the microarray study**

|  | **HD patients**  **(9)** | **Healthy subjects**  **(8)** | **Psychiatric patient**  **(5)** |
| --- | --- | --- | --- |
| Age, y  Mean ± SD | 48.16±8.5 | 49,17 ± 11,79 | 50,25 ± 11,47 |
| M/F | 4/5 | 4/4 | 3/2 |
| CAG  Mean ± SD | 42 | - | - |
| UHDRS-TMS  Mean ± SD | 24.4±20.06 | - | - |
| Disease duration  Mean ± SD | 4.43±2.80 | - | - |
