# Supplementary figures and images for: Circulating hsa-miR-323b-3p in Huntington's Disease: A Pilot Study
Source: Front Neurol. 2021 May 5;12:657973. doi: 10.3389/fneur.2021.657973 (PMC8131841; doi:10.3389/fneur.2021.657973)

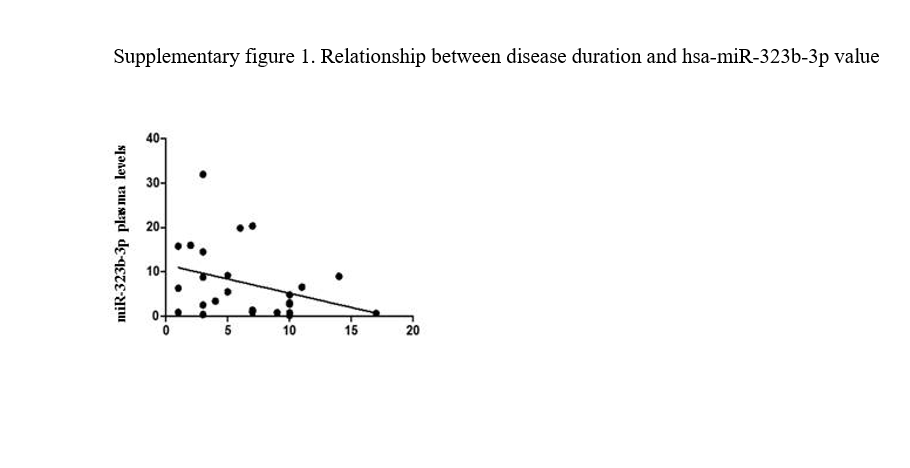

Supplement: Supplementary file 5 [file Image_1.tif]
